# Invisible threats: environmental asbestos exposure and water contamination in a former mining area of Northeastern Brazil

**DOI:** 10.1590/0102-311XEN271425

**Published:** 2026-09-18

**Authors:** Orleane Souza de Brito, Matheus Felipe Manduca Pereira, Carmen Sofia Murilhas Cardoso de Lima, Gabriel Abelha Carrijo-Gonçalves

**Affiliations:** 1 Departamento de Engenharia Sanitária e Ambiental, Instituto Federal de Educação, Ciências e Tecnologia da Bahia, Ilhéus, Brasil.; 2 Departamento de Engenharia Ambiental, Instituto Federal de Educação, Ciências e Tecnologia da Bahia, Vitória da Conquista, Brasil.; 3 Instituto Superior Tecnológico, Universidade de Lisboa, Lisboa, Portugal.; 4 Escola Politécnica, Universidade de São Paulo, São Paulo, Brasil.

**Keywords:** Environmental Monitoring, Environmental Health, Water Pollution, Asbestos, Chrysotile, Monitoramento Ambiental, Saúde Ambiental, Contaminação da Água, Amianto, Crisotila, Monitoreo del Ambiente, Salud Ambiental, Contaminación del Agua, Amianto, Crisotilo

## Abstract

Asbestos, a mineral fiber historically used in construction and industry due to its durability and resistance, is recognized as a Group 1 carcinogen by the International Agency for Research on Cancer (IARC), based on sufficient evidence of carcinogenicity through inhalation exposure. Although chrysotile asbestos was banned in Brazil in 2017, former mining areas remain major environmental liabilities, potentially sustaining long-term contamination of surrounding ecosystems. This study aimed to identify and characterize the qualitative presence of chrysotile asbestos fibers in surface waters and sediments in Bom Jesus da Serra, Bahia, Northeastern Brazil, where the São Félix mine operated for nearly three decades without subsequent remediation. Between August and November 2022, eight sampling sites were monitored across five field campaigns. Water and sediment samples were collected and analyzed for physicochemical parameters according to ABNT and CETESB standards, while asbestos fibers were detected and confirmed using phase-contrast microscopy, scanning electron microscopy coupled with energy-dispersive spectroscopy, and transmission electron microscopy. Chrysotile fibers were consistently detected in water and sediment samples from all sites, with higher accumulation in sediments, indicating persistent contamination of aquatic ecosystems. Most conventional water quality indicators complied with Brazilian standards. However, asbestos fibers were not quantified using regulatory metrics, preventing comparison with international guidelines and limiting exposure assessment. Their recurrent qualitative detection indicates environmental persistence and supports continued monitoring and future quantitative investigations to inform public health strategies in vulnerable communities.

## Abstract (pt)

O amianto, uma fibra mineral usada na construção civil e na indústria dado sua durabilidade e resistência, é reconhecido como carcinógeno do Grupo 1 pela Agência Internacional de Pesquisa em Câncer devido a evidências suficientes de sua carcinogenicidade através da exposição à inalação. Embora o Brasil tenha proibido o amianto crisotila em 2017, antigas áreas de mineração ainda oferecem grandes riscos ambientais, potencialmente sustentando a contaminação à longo prazo dos ecossistemas ao seu redor. Este estudo buscou identificar e caracterizar a presença qualitativa de fibras de amianto crisotila em águas superficiais e sedimentos em Bom Jesus da Serra, Bahia, Nordeste do Brasil, onde a mina São Félix operou por quase três décadas sem remediação subsequente. Entre agosto e novembro de 2022, oito locais de amostragem foram monitorados em cinco campanhas de campo. Amostras de água e sedimentos foram coletadas e analisadas acerca de seus parâmetros físicos e químicos seguindo os padrões ABNT e CETESB, enquanto fibras de amianto foram detectadas e confirmadas por microscopia de contraste de fase, microscopia eletrônica de varredura com espectroscopia de energia dispersiva e microscopia eletrônica de transmissão. Fibras de crisótilo foram consistentemente encontradas em amostras de água e em sedimentos em todos os locais, com maior acúmulo nos sedimentos, indicando contaminação persistente dos ecossistemas aquáticos. A maioria dos indicadores convencionais de qualidade da água estava em conformidade com as normas brasileiras. No entanto, as fibras de amianto não foram quantificadas usando métricas regulatórias, impedindo a comparação com diretrizes internacionais ou avaliação de exposição. Sua detecção qualitativa recorrente indica sua persistência ambiental, apoia o monitoramento contínuo e sugere futuras investigações quantitativas voltadas a estratégias de saúde pública em comunidades vulneráveis.

## Abstract (es)

El amianto, una fibra mineral utilizada en la construcción civil y la industria debido a su durabilidad y resistencia, está clasificado como carcinógeno del Grupo 1 por la Agencia Internacional de Investigación en Cáncer debido a evidencias suficientes de su carcinogenicidad a través de la exposición por inhalación. Aunque Brasil prohibió el amianto crisotilo en 2017, las antiguas zonas mineras aún representan grandes riesgos ambientales, con el potencial de mantener la contaminación a largo plazo de los ecosistemas circundantes. Este estudio tuvo como objetivo identificar y caracterizar la presencia cualitativa de fibras de amianto crisotilo en aguas superficiales y sedimentos en Bom Jesus da Serra, Bahía, Nordeste de Brasil, donde la mina São Félix operó durante casi tres décadas sin remediación posterior. Entre agosto y noviembre de 2022, se realizó un monitoreo de ocho puntos de muestreo en cinco campañas de campo. Se recogieron muestras de agua y sedimentos y se analizaron sus parámetros físicos y químicos según las normas ABNT y CETESB, mientras que las fibras de amianto se detectaron y confirmaron mediante microscopía de contraste de fase, microscopía electrónica de barrido con espectroscopia de energía dispersiva y microscopía electrónica de transmisión. Se encontraron fibras de crisotilo de forma consistente en muestras de agua y en sedimentos en todos los puntos, con una mayor acumulación en los sedimentos, lo que indica una contaminación persistente de los ecosistemas acuáticos. La mayoría de los indicadores convencionales de calidad del agua cumplían con las normas brasileñas. Sin embargo, las fibras de amianto no se han cuantificado a través de parámetros reglamentarios, lo que impide la comparación con las directrices internacionales o la evaluación de la exposición. Su detección cualitativa recurrente indica su persistencia ambiental, respalda el monitoreo continuo y sugiere futuras investigaciones cuantitativas centradas en estrategias de salud pública en comunidades vulnerables.

## Introduction

Environmental contamination by persistent toxic minerals remains one of the most pressing challenges to global public health ^1^ ^,^ ^2^. Among these, asbestos stands out for its dual legacy of industrial utility and irreversible harm. Classified by the International Agency for Research on Cancer (IARC) as a Group 1 carcinogen, asbestos is unequivocally associated with asbestosis, lung cancer, and mesothelioma, based on sufficient evidence of carcinogenicity through inhalation exposure ^3^. Despite international bans and growing evidence of its toxicity, asbestos remains a contaminant of concern due to its environmental persistence and the long latency period of asbestos-related diseases ^4^ ^,^ ^5^ ^,^ ^6^.

Although the carcinogenicity of asbestos via inhalation is well established, regulatory and scientific perspectives differ regarding potential risks associated with ingestion through drinking water. The World Health Organization (WHO) does not establish a guideline value for asbestos in drinking water, citing a lack of consistent epidemiological evidence linking fiber ingestion to cancer development. In contrast, the United States Environmental Protection Agency (USEPA), under the *Safe Drinking Water Act* ^7^, has established a maximum contaminant level (MCL) of 7 million fibers per liter (7MFL) for fibers longer than 10µm.

In Brazil, current water quality regulations, including *Resolution n. 357/2005* ^8^, by the Brazilian National Environmental Council (CONAMA, acronym in Portuguese), and drinking water standards issued by the Brazilian Ministry of Health, do not establish specific limits for asbestos fibers in water ^9^ ^,^ ^10^ ^,^ ^11^. In the absence of national regulatory thresholds for this contaminant, international reference frameworks, such as those proposed by WHO and USEPA, provide relevant comparative parameters for contextualizing environmental findings. These differing regulatory approaches reflect ongoing scientific uncertainties regarding the health effects of asbestos ingestion and reinforce the importance of site-specific investigations in historically impacted areas.

Historically, asbestos was valued for its thermal resistance, tensile strength, and chemical stability, leading to its extensive use throughout the 20th century in construction materials, insulation, and industrial products ^6^. The widespread incorporation of asbestos into the built environment resulted in global contamination extending far beyond occupational settings ^5^. Even after the discontinuation of asbestos use, degraded structures, waste deposits, and abandoned mining sites continue to release fibers into the air, soil, and water, posing diffuse risks to populations with no direct connection to industrial activities ^12^ ^,^ ^13^ ^,^ ^14^.

In recent studies, Macher et al. ^15^ reported that exposure to asbestos-contaminated water can induce toxic stress in plants, disrupting key physiological processes such as photosynthesis, nutrient absorption, and seed germination. The presence of asbestos fibers may also interfere with cellular division and metabolic activity, ultimately impairing plant growth and reproduction. These effects compromise overall plant vitality and increase vulnerability to pathogens and pests, particularly under additional environmental stressors. Therefore, the presence of asbestos fibers in the environment may negatively impact ecosystems.

Investigations conducted in Sibaté and Cartagena (Colombia) have expanded the understanding of environmental and non-occupational asbestos exposure in Latin America ^16^ ^,^ ^17^ ^,^ ^18^. These studies revealed a complex interaction between industrial legacy, social vulnerability, and systemic public health failures. In Sibaté, a municipality that hosted the country’s first asbestos-cement plant, researchers confirmed the presence of a pleural mesothelioma cluster, with age-adjusted incidence rates reaching 3.1 cases per 100,000 person-years among men and 1.6 among women ^16^. Strikingly, most diagnosed individuals had no history of occupational exposure, indicating that environmental, domestic, and para-occupational pathways were the primary determinants of disease occurrence.

One of the most critical mechanisms identified in Sibaté was the role of children as inadvertent vectors of asbestos contamination. During the 1980s and 1990s, former flooded areas were filled with friable and non-friable asbestos-containing industrial waste derived from asbestos-cement production ^19^. These unprotected landfills became informal recreational areas for children and adolescents, who frequently played directly on contaminated soils. Microscopic chrysotile and crocidolite fibers adhered to their clothing and footwear and were subsequently being transported into their homes. This process created a secondary, domestic exposure pathway analogous to para-occupational contamination historically observed among family members of asbestos workers ^16^. The handling and washing of contaminated garments further aerosolized fibers indoors, exposing parents and siblings. Agent-based modeling approaches later estimated that hundreds of individuals may have been exposed through this dynamic, underscoring how everyday social practices can transform children into unintentional conduits of environmental risk ^19^.

Field investigations revealed that the landfill materials consisted largely of industrial materials from asbestos-cement manufacturing, including debris, sludge, friable asbestos dust, broken asbestos-cement pipes, and construction fragments. These hazardous residues were historically deposited to drain and stabilize areas impacted by contamination from the El Muña reservoir and were later compacted to form the base for public infrastructure, including a football stadium and a school. Residents also reported using the same waste materials to level floors and reinforce the foundations of their homes, indicating widespread and poorly delimited urban contamination. The coexistence of chrysotile and crocidolite fibers, which are associated with high carcinogenic potency, reinforces the severity of long-term environmental exposure in the municipality of Sibaté ^17^ ^,^ ^20^.

Research conducted in Cartagena ^18^ broadened the discussion by emphasizing the structural role of poverty and social practices in sustaining chronic exposure scenarios. In low- and middle-income neighborhoods, 87.5% of households had asbestos-cement roofing. Environmental assessments detected asbestos fibers in 90% of outdoor dust samples and in 85% of rainwater samples collected from rooftops, demonstrating pervasive environmental contamination within domestic settings ^18^. Although more than half of residents recognized the health risks associated with asbestos, economic constraints compelled them to continue unsafe maintenance practices, such as sanding, washing, reusing, or informally discarding deteriorated roofing materials. These findings illustrate how exposure is not merely a consequence of historical industrial activity, but also results from ongoing structural vulnerabilities that limit access to safer housing alternatives.

In Brazil, the environmental and health implications of asbestos are particularly complex. For decades, the country was one of the world’s largest producers and exporters of chrysotile, the serpentine form of asbestos ^11^. Although the Brazilian Supreme Federal Court banned its use and commercialization in 2017, numerous sites remain without remediation, especially in rural or economically vulnerable municipalities. These sites continue to represent chronic sources of contamination, affecting water bodies and local ecosystems, creating pathways for non-occupational human exposure ^13^ ^,^ ^21^.

The environmental persistence of asbestos fibers is enhanced by their mineralogical properties. Resistant to degradation and capable of remaining suspended or being re-deposited over long periods, chrysotile fibers may be transported by wind and hydrological processes, reaching distant areas ^21^. In semi-arid regions, where low humidity and intense winds prevail, resuspension of contaminated sediments further amplifies the potential for inhalation and waterborne exposure. This scenario is further aggravated by the scarcity of environmental monitoring outside occupational settings, which limits the understanding of asbestos ecological dynamics and their health implications in post-mining environments ^12^ ^,^ ^13^ ^,^ ^22^.

The municipality of Bom Jesus da Serra, located in Bahia State, Northeastern Brazil, is a critical case study in this context. The São Félix mine, active from the late 1930s until 1967, was the first chrysotile asbestos mine in Brazil. Decades after its closure, the area remains without remediation, with visible erosion, tailing deposits, and sediment dispersion across the local hydrological network ^10^. Historical evidence and community reports suggest that asbestos residues persist in soil and surface water, but no systematic environmental assessment had previously been conducted.

This lack of empirical data represents a significant knowledge gap in the understanding of non-occupational asbestos exposure in Brazil. Despite the existence of public health monitoring programs such as Datamianto, which track asbestos-related diseases, environmental data remains poorly integrated into these systems ^11^. Establishing an environmental baseline for asbestos contamination in post-mining areas is essential for developing preventive strategies and guiding remediation and health monitoring efforts.

Therefore, this study aimed to identify and characterize the presence of chrysotile asbestos fibers in surface water and bottom sediments in Bom Jesus da Serra, near the São Félix mining area. By combining field monitoring and high-resolution microscopy techniques, this research seeks to provide the first empirical evidence of multicomponent asbestos contamination in this region, contributing to the national and global discussion on environmental health risks associated with legacy asbestos mining.

## Materials and methods

### Study area and sampling design

**Figure 1:**
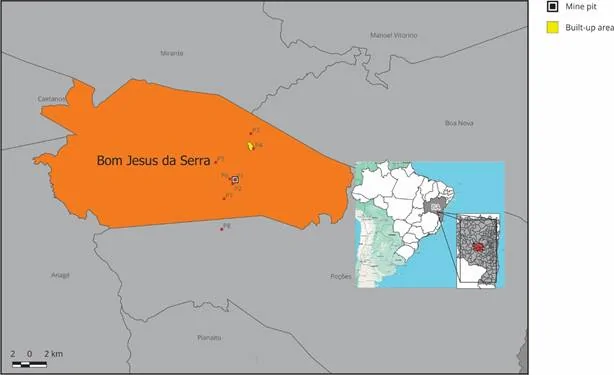
Geographical distribution of the study area with location map and sampling sites in the municipality of Bom Jesus da Serra, Bahia State, and the former São Félix chrysotile mine.

Source: prepared by the authors, based on data from Brazilian Institute of Geography and Statistics (IBGE; https://www.ibge.gov.br/).

Note: Geographic Coordination System, DATUM SIRGAS 2000 - EPSG 31984, scale 1:180,000.

This study was conducted in the municipality of Bom Jesus da Serra, located in the southwestern region of Bahia State, Northeastern Brazil (14º24’S; 40º31’W), as presented at Figure 1. The municipality is located within the Caatinga biome and is also characterized by a semi-arid BSh climate according to the Köppen classification. Mean annual rainfall is approximately 540mm, with temperatures ranging from 19.2ºC to 23.4ºC ^23^. The municipality covers approximately 468km^2^ and has a population of 9,768 inhabitants, reflecting socioeconomic vulnerability and limited infrastructure ^24^.

Sampling sites were distributed across lentic water bodies located near the deactivated São Félix asbestos mine, which operated from 1936 to 1967 and left approximately 280 hectares of environmentally degraded land ^9^. Eight sampling locations were selected based on hydrological relevance, community use, and proximity to the former mining structures. Each site comprised a water sampling point (WP1-WP8) and a corresponding bottom-sediment point (SP1-SP8), as presented in Figure 1 and summarized at Table 1. Geographic coordinates and elevations were recorded using a Garmin GPS unit, WGS84 system (https://www.garmin.com), to ensure spatial reproducibility.

**Table 1:** Location of water and sediment sampling points.

Water point	Sediment point	Location	Geographic coordinates	Elevation (m)	Distance from mine (km)
WP1	SP1	Mine pit	S 14º24’31.8” / W 40º31’23.1”	634	0.000
WP2	SP2	Mine cauldron	S 14º24’48.6” / W 40º31’3.9”	675	0.562
WP3	SP3	Ponte reservoir	S 14º21’31.8” / W 40º30’19.8”	595	5.860
WP4	SP4	Lagoa square	S 14º22’30.1” / W 40º30’9.2”	634	4.346
WP5	SP5	Cai n’Água dam	S 14º24’30” / W 40º31’42.6”	646	3.077
WP6	SP6	Water reservoir	S 14º23’24.6” / W 40º32’38.1”	639	0.600
WP7	SP7	Jurema	S 14º25’49.1” / W 40º32’3.5”	656	2.658
WP8	SP8	Patos lagoon	S 14º27’47.9” / W 40º32’14.5”	724	6.199

Field campaigns were conducted over a three-month period on August 12th, September 2nd and 26th, October 21st, and November 4th, 2022. At each sampling event, triplicate water samples were collected from all eight sites, yielding a total of 120 water samples. In parallel, 40 bottom-sediment samples were collected during the same period. All sampling procedures adhered to standard environmental monitoring practices, considering accessibility, depth, and environmental conditions at each site.

Daily rainfall during the sampling period was measured using an Incoterm 0-150mm manual pluviometer (https://www.incoterm.com.br), operated by a trained local resident. Each milliliter recorded by the device corresponded to 1L/m^2^ of precipitation. Data were used to contextualize hydrological conditions and evaluate temporal variability in local rainfall.

### Water and sediment sample collections

Water and bottom sediment samples were collected following the procedures established in the standard NBR 9898 (1987) by the Brazilian Association of Technical Standards (ABNT, acronym in Portuguese) for environmental sampling ^25^ and the National Sampling and Preservation Guide (2011) by the São Paulo State Environmental Company (CETESB, acronym in Portuguese) ^26^.

At each sampling point, surface water samples (0.5L-1.0L) were collected at a depth of 15cm-30cm using high-density polyethylene (HDPE) bottles previously cleaned with 65% nitric acid and rinsed with deionized water to prevent cross-contamination. Due to accessibility constraints, a Van Dorn horizontal sampler was also employed to collect water samples. Sediment samples (approximately 300g) were collected using a stainless-steel Ekman-type sediment grabber adapted by professionals of the Bahia State Federal Institute of Education, Science and Technology (IFBA, acronym in Portuguese), targeting the fine-grained fraction (< 63µm), which is known to favor the retention and accumulation of fibrous particles such as chrysotile.

Immediately after collection, water and sediment samples were stored in insulated thermal containers with reusable ice packs to maintain internal temperatures between 2ºC and 8ºC. Temperature conditions were monitored throughout transportation to ensure compliance with preservation requirements. The elapsed time between field collection and laboratory receipt did not exceed six hours, minimizing potential physicochemical or microbiological alterations. Upon arrival at the laboratory, water samples were refrigerated, and sediment samples were prepared for treatment.

Sediments were filtered through quantitative filter paper placed in glass funnels for 24h and subsequently dried in an oven at 70ºC for 48h to eliminate retained moisture. Water samples intended for microscopic analysis were fully evaporated in a muffle furnace at 300ºC to concentrate residues. After cooling in a desiccator, the resulting residue was removed using a stainless-steel spatula and mounted onto glass slides for further analysis.

All procedures followed established Brazilian standards for environmental monitoring and laboratory safety. The study design ensured representativeness of environmental compartments and methodological traceability, providing reliable baseline data to support future public health and remediation initiatives.

### Physicochemical analyses of water

Physicochemical evaluations included pH, turbidity, temperature, dissolved oxygen, electrical conductivity, salinity, and total dissolved solids (TDS). pH measurements were performed using a Hanna benchtop pH meter (https://hannainst.com.br). Electrical conductivity was measured using a Tecnopon MCA-150 conductivity meter (https://www.tecnopon.com.br), which was automatically calibrated before each analysis. Temperature and dissolved oxygen were measured in situ using portable field instruments. Turbidity was determined using a portable turbidimeter. Salinity and TDS were estimated using the Qualigraf software (https://qualigraf.funceme.br/), based on the relationship between conductivity and dissolved solid concentration.

### Microbiological analyses

Microbiological analyses were performed using the Colilert method with the Quanti-Tray system (https://www.idexx.com), followed by incubation for 24h at 35ºC (± 0.5ºC) and subsequent reading of positive wells under ultraviolet light, in accordance with the manufacturer’s protocol. Although the method enables quantitative estimation expressed as the most probable number per 100mL (MPN/100mL), results were systematized for the purpose of determining the presence or absence of total coliforms and *Escherichia coli*, consistent with the monitoring objectives established at the time of sampling. Accordingly, the data presented in this study refer to the qualitative classification of the analyzed samples.

### Microscopic identification of fibrous material

Initial screening for fibrous particles was conducted using a BEL stereoscopic binocular microscope with 45x magnification (https://www.belequipamentos.com.br). Samples with suspected mineral fibers underwent detailed examination using scanning electron microscopy (SEM) equipped with energy-dispersive spectroscopy (EDS) on a Tescan Vega 3 SEM (https://tescan.com), enabling morphological and elemental characterization at the Electrochemistry and Corrosion Laboratory, Department of Chemical Engineering, Engineering School of University of São Paulo (Brazil). Complementary analyses using transmission electron microscopy (TEM) and polarized light microscopy (PLM) were performed at Eurofins Laboratory (Portugal), accredited under ISO/IEC 17025 standard by the International Organization for Standardization ^27^, for definitive chrysotile fibers identification.

## Results

### Environmental and territorial characterization

The study area is located in a semi-arid rural context where surface water bodies are routinely used for domestic supply, livestock watering, and small-scale agriculture, increasing the potential for continuous human-environment interaction. The historical operation of the São Félix asbestos mine, followed by the absence of post-closure remediation, has led to the accumulation of asbestos-containing residues in soils and their proximity to surface watercourses, constituting long-term environmental liabilities ^21^ ^,^ ^28^.

During the five field campaigns (C1-C5) conducted between August and November 2022, environmental alterations were observed, including elevated turbidity, mineral-rich sediments, and degradation of riparian vegetation. These features are consistent with post-asbestos mining environments, where fibrous residues persist and remain susceptible to remobilization over extended periods ^10^ ^,^ ^11^ ^,^ ^22^.

### Hydrological conditions

Rainfall monitoring indicated predominantly dry conditions throughout the study period, with only a few isolated precipitation events, consistent with the semi-arid climate of the region ^23^. The first and second sampling campaigns were conducted under hydrologically stable conditions, with no significant antecedent rainfall and only negligible precipitation (≈ 1mm) recorded afterward. In contrast, the third campaign, conducted on September 26, 2022, coincided with an intense rainfall event approximately 55mm, likely promoting surface runoff, sediment resuspension, and enhanced mobilization of fine particles accumulated in streambeds.

The fourth and fifth campaigns occurred, respectively, after and before moderate rainfall events of approximately 10mm and 20mm, with minor additional precipitation recorded shortly thereafter. These temporal variations enabled the assessment of water and sediment quality under both stable and post-runoff conditions.

### Physicochemical and microbiological water quality

Electrical conductivity values exhibited marked spatial heterogeneity across the eight sampling points (Table 2). Sites located closer to the former mining area, especially WP1, WP3, and WP4, consistently presented elevated conductivity values, reaching approximately 3,000µS/cm in some campaigns. In contrast, sites farther from the mining area showed substantially lower values.

**Table 2:** Physicochemical parameters and water body classification of surface water samples (WP1-WP8) collected during the 2022 monitoring campaigns (C1-C5).

Water samples/Campaigns	Electrical conductivity at 25ºC (µS/cm)	TDS (mg/L)	Water body classification	pH	Turbidity (NTU)	Temperature (ºC)	DO (mg/L)
WP1							
C1	2,103 ± 16	1,366	Brackish water	8.7 ± 0.1	0.20 ± 0.08	24.1 ± 0.5	6.43 ± 0.06
C2	1,898 ± 19	1,233	Brackish water	8.8 ± 0.1	1.49 ± 0.28	19.8 ± 0.3	8.35 ± 0.06
C3	2,075 ± 19	1,348	Brackish water	8.8 ± 0.1	0.68 ± 0.11	24.5 ± 0.1	7.71 ± 0.01
C4	2,087 ± 59	1,357	Brackish water	9.0 ± 0.1	0.86 ± 0.33	25.4 ± 0.4	11.34 ± 0.06
C5	2,108 ± 29	1,370	Brackish water	8.8 ± 0.1	1.08 ± 0.11	22.7 ± 0.3	4.03 ± 0.57
WP2							
C1	79 ± 2	51	Fresh water	6.2 ± 0.1	0.06 ± 0.02	26.6 ± 1.3	6.38 ± 0.07
C2	71 ± 2	46	Fresh water	8.6 ± 0.2	0.45 ± 0.20	22.6 ± 0.1	7.87 ± 0.05
C3	67 ± 1	44	Fresh water	8.7 ± 0.2	0.34 ± 0.03	25,2 ± 0.5	0.25 ± 0.06
C4	77 ± 3	49	Fresh water	9.0 ± 0.2	0.43 ± 0.02	27.2 ± 0.9	0.51 ± 0.11
C5	82 ± 2	53	Fresh water	8.0 ± 0.2	4.45 ± 0.07	22.9 ± 0.2	1.14 ± 0.67
WP3							
C1	1,880 ± 15	1,221	Brackish water	7.9 ± 0.1	0.35 ± 0.05	27.1 ± 1.5	7.31 ± 0.17
C2	1,642 ± 11	1,067	Brackish water	8.0 ± 0.1	2.45 ± 0.21	21.3 ± 0.6	8.12 ± 0.07
C3	1,769 ± 39	1,150	Brackish water	8.2 ± 0.2	6.60 ± 2.40	25.8 ± 0.6	0.61 ± 0.09
C4	1,907 ± 36	1,239	Brackish water	7.8 ± 0.2	1.97 ± 1.32	25.0 ± 0.6	3.79 ± 0.18
C5	1,964 ± 18	1,277	Brackish water	8.0 ± 0.1	0.43 ± 0.04	22.6 ± 0.2	3.21 ± 0.34
WP4							
C1	2,755 ± 7	1,772	Saline water	8.9 ± 0.1	10.70 ± 0.46	27.0 ± 0.8	7.85 ± 0.07
C2	2,545 ± 21	1,622	Saline water	8.9 ± 0.1	104.00 ± 16.97	23.0 ± 0.4	7.90 ± 0.05
C3	2,240 ± 42	1,437	Brackish water	8.7 ± 0.1	1.97 ± 0.35	25.6 ± 0.4	2.97 ± 0.05
C4	2,310 ± 42	1,531	Saline water	8.6 ± 0.2	6.46 ± 0.23	26.8 ± 0.6	6.02 ± 0.26
C5	2,640 ± 10	1,716	Saline water	8.8 ± 0.1	5.65 ± 4.60	22.0 ± 0.1	4.57 ± 0.61
WP5							
C1	296 ± 4	192	Fresh water	8.3 ± 0.2	0.06 ± 0.03	28.0 ± 0.1	11.53 ± 0.25
C2	309 ± 26	200	Fresh water	7.9 ± 0.4	8.30 ± 1.27	21.9 ± 0.6	8.00 ± 0.15
C3	382 ± 4	248	Fresh water	8.5 ± 0.2	9.70 ± 0.28	25.3 ± 0.8	0.69 ± 0.05
C4	377 ± 5	244	Fresh water	8.1 ± 0.1	8.30 ± 1.13	25.9 ± 0.8	2.87 ± 0.40
C5	408 ± 2	265	Fresh water	8.2 ± 0.1	6,30 ± 0.57	23.5 ± 0.1	2.34 ± 0.45
WP6							
C1	356 ± 5	231	Fresh water	8.0 ± 0.1	8.45 ± 3.04	30.1 ± 0.5	7.16 ± 0.13
C2	257 ± 4	167	Fresh water	7.5 ± 0.1	1.81 ± 0.18	22.1 ± 0.1	8.02 ± 0.01
C3	293 ± 3	190	Fresh water	8.3 ± 0.1	1.69 ± 1.43	26.5 ± 0.6	0.91 ± 0.14
C4	331 ± 1	215	Fresh water	7.7 ± 0.1	4.50 ± 0.10	27.6 ± 0.3	3.63 ± 0.33
C5	335 ± 1	218	Fresh water	7.6 ± 0.3	0.91 ± 0.28	22.1 ± 0.1	3.92 ± 0.20
WP7							
C1	743 ± 7	483	Fresh water	7.8 ± 0.1	45.50 ± 2.12	27.9 ± 0.1	7.39 ± 0.09
C2	780 ± 22	517	Brackish water	7.7 ± 0.1	367.50 ± 14.85	23.4 ± 0.1	7.84 ± 0.02
C3	681 ± 5	443	Fresh water	7.9 ± 0.2	198.33 ± 6.03	27.9 ± 0.5	3.13 ± 0.55
C4	1,252 ± 82	814	Brackish water	7.9 ± 0.2	721.00 ± 33.94	28.5 ± 0.7	4.73 ± 0.17
C5	*	*	*	*	*	*	*
WP8							
C1	168 ± 3	107	Fresh water	8.1 ± 0.1	10.25 ± 0.35	25.1 ± 0.1	7.67 ± 0.05
C2	145 ± 4	94	Fresh water	7.6 ± 0.1	105.50 ± 0.71	22.0 ± 0.3	8.03 ± 0.04
C3	159 ± 4	103	Fresh water	7.8 ± 0.1	113.50 ± 10.61	24.3 ± 0.4	3.06 ± 0.19
C4	173 ± 3	112	Fresh water	7.7± 0.4	119.33 ± 11.93	27.0 ± 0.4	4.13 ± 0.71
C5	183 ± 1	118	Fresh water	7.6 ± 0.3	130.50 ± 0.71	21.5 ± 0.8	2.96 ± 0.44

DO: dissolves oxygen; NTU: nephelometric turbidity unit; TDS: total dissolved soldis.

* The data line for WP7 does not extend to C5 because there was no water source available.

TDS followed the same spatial distribution observed for conductivity (Table 2), reinforcing the strong relationship between these parameters. Water classification ranged from fresh to brackish and saline, depending on location and campaign. Turbidity values were particularly elevated at WP4 and WP7, reaching 104NTU and 721NTU, respectively, during specific campaigns. Dissolved oxygen levels showed temporal fluctuations, with some values below 1mg/L at certain sites following rainfall events.

Microbiological analyses detected total coliforms and *E. coli* in surface water samples from several sampling points (Table 3), indicating localized fecal contamination associated with human and animal activities.

**Table 3:** Presence of total coliforms and *Escherichia coli* in surface water samples analyzed using the Colilert method during the 2022 monitoring campaigns (C1-C5).

Water samples	C1	C2	C3	C4	C5
WP1	Total coliforms	Absence of coliforms	Total coliforms	Total coliforms	Total coliforms
WP2	Total coliforms	Total coliforms	Total coliforms	Total coliforms	Total coliforms
WP3	*E. coli.*	Absence of coliforms	*E. coli.*	*E. coli.*	*E. coli.*
WP4	Total coliforms	Absence of coliforms	*E. coli.*	Total coliforms	*E. coli.*
WP5	Total coliforms	Total coliforms	*E. coli.*	Total coliforms	*E. coli.*
WP6	*E. coli.*	Total coliforms	*E. coli.*	Total coliforms	*E. coli.*
WP7	*E. coli.*	Total coliforms	*E. coli.*	Total coliforms	*
WP8	*E. coli.*	Total coliforms	*E. coli.*	Total coliforms	Total coliforms

Color scheme used to indicate microbiological results: red (presence of *Escherichia coli*); yellow (presence of total coliforms); green (absence of coliforms); white (no sample analyzed).

* The data line for WP7 does not extend to C5 because there was no water source available.

### Microscopic identification of fibrous minerals

**Figure 2:**
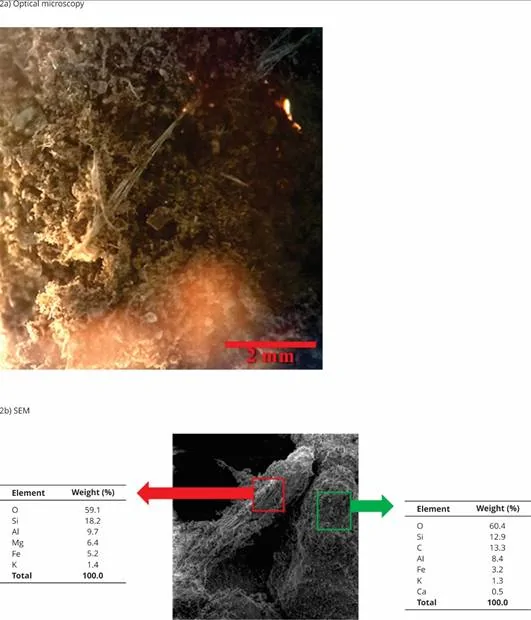
Visualization of asbestos-like fibers using optical microscopy and scanning electron microscopy (SEM) imaging with elemental composition of asbestos-like fibrous particles detected in environmental samples by energy-dispersive X-ray spectroscopy (EDS).

The initial stereoscopic screening revealed elongated and fibrillar particles predominantly associated with fine-grained sediment fractions, especially at sites located downstream of the former São Félix mine (Figure 2). Comparison of the images obtained by stereoscopic microscopy and SEM revealed similar fibrous morphologies. EDS analysis indicated the presence of oxygen (O), silicon (Si), magnesium (Mg), aluminum (Al), and carbon (C), with a chemical composition consistent with chrysotile/asbestos minerals ^28^.

Al: aluminum; C: carbon; Ca: calcium; Fe: iron; K: potassium; Mg: magnesium; Si: silicon.

The recurrent detection of fibrous material in both sediment and water samples made it necessary to determine the specific fiber type present in the study area. For this purpose, two sediment samples were submitted for analysis at the Eurofins Laboratory (France), where they were examined using PLM and TEM. The combined results of these analyses confirmed that the fibers identified in the samples corresponded to chrysotile asbestos. In addition to fiber identification, elemental analysis revealed a predominance of Mg (54%) and Si (40%), with minor contributions of iron (Fe) (1%).

## Discussion

### Hydrological variability and contaminant mobilization

The temporal relationship between rainfall events and sampling campaigns suggests that episodic precipitation is critical in sediment resuspension and contaminant redistribution. The intense rainfall recorded before C3 (~ 55mm) likely contributed to increased turbidity and dissolved solids through surface runoff. In semi-arid environments, prolonged dry periods favor accumulation of fine particles and contaminants in streambeds, while episodic rainfall triggers redistribution pulses, reinforcing the dynamic nature of legacy contamination ^29^.

The study area is located in a semi-arid rural context where surface water bodies are routinely used for domestic supply, livestock watering, and small-scale agriculture, increasing the potential for continuous human-environment interaction. The historical operation of the São Félix asbestos mine, followed by the absence of post-closure remediation, has led to the accumulation of asbestos-containing residues in soils and their proximity to surface watercourses, constituting long-term environmental liabilities ^21^ ^,^ ^28^. The overlap between contaminated environments and routine human use characterizes a scenario of chronic, low-intensity exposure, typical of legacy mining areas and relevant to environmental health surveillance ^30^.

Similar patterns of environmental persistence and redistribution have been documented in Sibaté, where asbestos-containing residues deposited in urban landfills remained environmentally active decades after industrial closure. There, seasonal hydrological processes and surface disturbances contributed to continued exposure scenarios even in the absence of ongoing industrial activity ^16^ ^,^ ^19^. The São Felix case aligns with this evidence, suggesting that climatic and geomorphological processes are critical determinants of long-term exposure in post-asbestos territories.

### Combined physicochemical and microbiological stressors

The pronounced heterogeneity in conductivity and TDS suggests localized enrichment of dissolved ions from mineral dissolution. These findings indicate sustained geochemical interactions between contaminated sediments and the water column, consistent with the long-term presence of asbestos-bearing materials in the environment ^13^ ^,^ ^21^ ^,^ ^29^.

Additional physicochemical parameters revealed further environmental stressors. Elevated turbidity and suspended solids were frequently observed near residue deposits, reflecting active sediment mobilization and increased potential for the transport of fibrous particles. Although pH values remained within near-neutral ranges, consistent with natural regional conditions, such values do not prevent mineral leaching or fiber persistence in aquatic systems ^29^.

Communities relying on surface waters located near mining residues may experience substantially different exposure conditions compared to those using water bodies farther from contaminated sites. Similar findings were reported in a study investigating soil contamination near a former vermiculite processing plant in Honolulu (United States) ^31^. The authors reported that, even four decades after closure, local wind dynamics continued to influence the the behavior of fibrous materials, resulting in spatial variability across different locations within the study area. This reinforces the importance of territorially differentiated monitoring and management strategies rather than uniform regulatory approaches ^30^.

These findings are consistent with rural settings characterized by limited sanitation infrastructure and direct use of natural water bodies for multiple purposes ^10^. The presence of microbiological contamination adds a critical dimension to the assessment of environmental quality and health risk. The coexistence of microbiological contamination, physicochemical alterations, and mineral residues amplifies environmental vulnerability. Elevated turbidity and suspended solids can facilitate the survival and transport of microorganisms, while reduced dissolved oxygen (DO) levels may reflect ecological degradation, particularly during the third campaign at WP2, WP3 WP5, and WP6 after intense rainfall (55mm). Together, these stressors create complex exposure scenarios in which chemical, physical, and biological factors interact ^10^ ^,^ ^13^ ^,^ ^29^.

### Sediments as long-term reservoirs and exposure amplifiers

Microscopic analyses provided evidence of the persistence of fibrous minerals in soils and sediments from the studied area several decades after the cessation of asbestos mining activities ^21^ ^,^ ^28^. The fibrous material exhibited a chemical composition distinct from that of the surrounding sediment matrix. Elemental analysis reveals a predominance of O, Si, Al, and Mg in relatively high proportions, which is consistent with the mineralogical signature expected for asbestos-related fibrous materials ^21^ ^,^ ^31^. This compositional distinction supports the interpretation that the observed fibers are not merely fragments of the local sediment but represent a distinct mineral phase.

The identification of well-defined fibrous bundles with high aspect ratios by SEM, coupled with elemental compositions compatible with asbestos-bearing minerals determined by EDS, indicates that these materials remain structurally intact and environmentally available. Similar persistence has been reported in international studies conducted in former asbestos mining regions, where fibers have been detected in sediments and soils decades after mining cessation, maintaining their potential for remobilization and exposure.

This elemental composition is consistent with the results obtained by scanning electron microscopy coupled with energy-dispersive spectroscopy (SEM-EDS). The observed Mg-Si dominance constitutes a well-established compositional fingerprint characteristic of chrysotile asbestos, reinforcing the reliability of the identification ^28^ ^,^ ^31^.

The preferential accumulation of fibrous materials in depositional environments highlights the role of sediments as long-term contamination reservoirs. In semi-arid regions such as the study area, episodic rainfall events and seasonal hydrological variability can intermittently disturb these reservoirs, promoting the resuspension and downstream transport of fibers. This dynamic reinforces the understanding that contamination in legacy mining areas is not static, but rather subject to periodic redistribution, which may temporarily increase exposure risks even in the absence of new sources.

From a public health perspective, the persistence of asbestos fibers in environmental matrices raises significant concerns regarding chronic exposure pathways. Unlike occupational exposure scenarios, which are often more easily identifiable and regulated, environmental exposure in rural settings tends to be diffuse, continuous, and socially invisible. The use of surface water bodies for domestic activities, livestock watering, and small-scale agriculture creates multiple opportunities for repeated contact with contaminated sediments and suspended particles, resulting in a scenario of low-dose, long-term exposure that is particularly challenging for conventional health surveillance systems ^16^ ^,^ ^19^ ^,^ ^30^.

The detection of fibrous minerals in sediments should not be interpreted solely as an environmental issue, but also as a relevant indicator of potential health risk, especially in contexts marked by socioeconomic vulnerability and limited access to alternative water sources ^18^. The environmental reservoir presented by sediments may subsequently contribute to domestic exposure, particularly among children who play in contaminated sites (as reported in Sibaté) and unintentionally transport fibers into households via clothing, a mechanism analogous to para-occupational exposure ^16^ ^,^ ^19^.

Although the Brazilian study area differs from Sibaté in terms of geomorphological configuration, the underlying mechanism is similar: the persistence of environmental reservoirs that remain socially accessible and environmentally mobile. In this sense, the results support the need for precautionary management strategies, continuous environmental surveillance, and the inclusion of legacy mining areas within broader public health policies aimed at reducing environmental inequalities and preventing chronic exposure to hazardous materials.

### Implications for environmental monitoring and public health

One of the most critical lessons derived from the Colombian cases concerns the limitations of passive surveillance systems. In Sibaté, official national health databases did not initially capture mesothelioma cases associated with environmental exposure. Only through active case-finding, medical record validation, and interdisciplinary investigation was the epidemiological cluster formally recognized. The Brazilian findings presented here underscore the importance of preventive governance before clinical manifestations emerge. The identification of persistent chrysotile in environmental matrices should not be interpreted as an isolated mineralogical finding, but as an early warning signal requiring institutional response.

The results of this study identified distinct zones of environmental risk, characterized by persistent physicochemical alterations, microbial contamination, and the presence of asbestos-compatible fibrous materials. These findings, when interpreted within a public health and territorial framework, emphasize the need for integrated approaches that combine environmental remediation, long-term monitoring, and health surveillance. Asbestos residues in sediments and soils, even decades after mine closure, demonstrate behavior consistent with other post-mining environments where fibrous minerals remain active and capable of remobilization ^13^.

Effective remediation of legacy asbestos contamination requires multiple, context-specific interventions. In aquatic systems with fine sediment accumulation, controlled dredging or sediment removal can reduce fiber reservoirs that act as secondary contamination sources. Such interventions have been considered in mining contexts to limit long-term particle resuspension, although methodological refinements are needed to minimize secondary dispersion during operation ^31^ ^,^ ^32^. Additionally, sediment capping, which involves placing clean material over contaminated deposits, has been documented as a strategy to immobilize contaminants and reduce their availability for transport during hydrological events.

Land-based measures are equally critical. Soil sealing and stabilization, including the use of geotextiles or hard surface covers, can reduce erosion and dust generation, thereby limiting the release of asbestos fibers into adjacent water bodies and the atmosphere ^29^. Phytostabilization and revegetation with native plant species are increasingly recognized for their capacity to enhance soil structure, reduce erosion, and improve ecological function, particularly in semi-arid environments where native vegetation contributes to sediment retention and bank stabilization ^33^.

Moreover, international guidance underscores the importance of integrating risk communication and community engagement into remediation planning. Populations living near contaminated sites often depend on surface water for domestic and agricultural purposes and may therefore experience cumulative exposures to chemical, biological, and physical stressors ^30^. Providing alternative water sources, clear signage for high-risk areas, and education on safe water use can mitigate chronic exposure even before structural remediation is fully implemented. Regulatory frameworks in many jurisdictions advocate for such phased approaches, combining environmental management with public health protection.

Environmental health surveillance should also be strengthened to monitor long-term outcomes. Establishing baseline health indicators and conducting periodic assessments of environmental and biological markers can help detect emerging impacts before they become clinically apparent. Asbestos exposure is associated with serious health outcomes, including mesothelioma and chronic respiratory diseases, emphasizing the importance of these surveillance systems ^34^.

In summary, the interplay between environmental contamination and human activities in former mining territories requires multidimensional and sustained responses. Combining physical remediation, ecological restoration, risk communication, and health surveillance aligns with best practices in environmental health and collective protection, providing a framework that integrates local needs, scientific evidence, and policy expectations.

## Conclusions

The findings of this study document the recurrent qualitative detection of asbestos fibers in water and sediment samples collected from an area historically impacted by chrysotile mining in northeastern Brazil. The persistence of fibrous material decades after mine closure indicates the continued presence of environmental liabilities associated with former extraction activities. Although the analytical approach adopted was qualitative, the consistent identification of fibers across monitoring campaigns suggests that legacy residues remain environmentally detectable in surface matrices.

The physicochemical and microbiological analyses contributed to a broader environmental characterization of the sampled sites. However, these parameters, when considered in isolation, do not establish a causal relationship with the presence of asbestos fibers, nor do they allow direct quantification of exposure or health risk. Accordingly, the results should be interpreted as evidence of environmental occurrence rather than as a direct assessment of human health risk.

It is important to recognize that international regulatory and scientific perspectives differ regarding the potential risks associated with asbestos ingestion through drinking water. The IARC classifies asbestos as a Group 1 carcinogen based on sufficient evidence for inhalation exposure, while evidence concerning ingestion remains inconclusive. The WHO has not established a guideline value for asbestos in drinking water, citing insufficient consistent evidence of carcinogenicity via ingestion. In contrast, the USEPA establishes a maximum contaminant level of 7MFL for fibers longer than 10µm.

As this investigation did not quantify fiber concentrations in terms of MFL or mass per sediment unit, comparisons with regulatory thresholds were not possible. Considering these differences among international regulatory frameworks, particularly between WHO and USEPA, the findings of this study should be interpreted as indicators of environmental concern and the need for continued monitoring, rather than as an isolated quantification of health risk associated with ingestion.

In this context, the study reinforces the importance of systematic environmental monitoring in territories with a history of asbestos mining, particularly where communities remain in proximity to former operational structures. Future investigations incorporating quantitative fiber analysis and integrated exposure assessment would enable more robust comparisons with international regulatory frameworks and contribute to evidence-based environmental management strategies.

By documenting the persistence and environmental mobility of asbestos fibers in non-occupational contexts, the findings provide a technical basis for public health actions aimed at reducing exposure, protecting vulnerable populations, and strengthening environmental governance in areas affected by legacy asbestos contamination.

Therefore, environmental health surveillance in post-mining territories should integrate: (i) long-term monitoring of sediments and surface waters; (ii) territorial mapping of exposure pathways; (iii) community risk communication; and (iv) strengthened epidemiological surveillance of asbestos-related diseases. The comparison with Sibaté and Cartagena illustrates that delayed recognition of environmental asbestos contamination can result in prolonged, socially invisible exposure, disproportionately affecting vulnerable populations.

## Data Availability

The research data are available upon request to the corresponding author.
